# Supplementary material for: S-Nitrosocysteine Modulates Nitrate-Mediated Redox Balance and Lipase Enzyme Activities in Food-Waste-Degrading Burkholderia vietnamiensis TVV75 to Deter Salt Stress
Source: Microorganisms. 2025 Nov 10;13(11):2559. doi: 10.3390/microorganisms13112559 (PMC12654293; doi:10.3390/microorganisms13112559)
Supplement: Supplementary file 1 [file microorganisms-13-02559-s001.zip › microorganisms-3956621-supplementary.pdf]

S-nitrosocysteine modulates nitrate-mediated redox balance and lipase enzyme activities in food-waste degrading *Burkholderia vietnamiensis* TVV75 to deter salt stress

Youn-Ji Woo[1†], Da-Sol Lee[1†], Ashim Kumar Das[1], Geum-Jin Lee[1], Bong-Gyu Mun[2] and Byung-Wook Yun[1,*]

[1]Department of Applied Biosciences, College of Agriculture and Life Sciences, Kyungpook National University, Daegu, 41566, South Korea

[2]Department of Environment and Biological Chemistry, Chungbuk National University, Republic of Korea

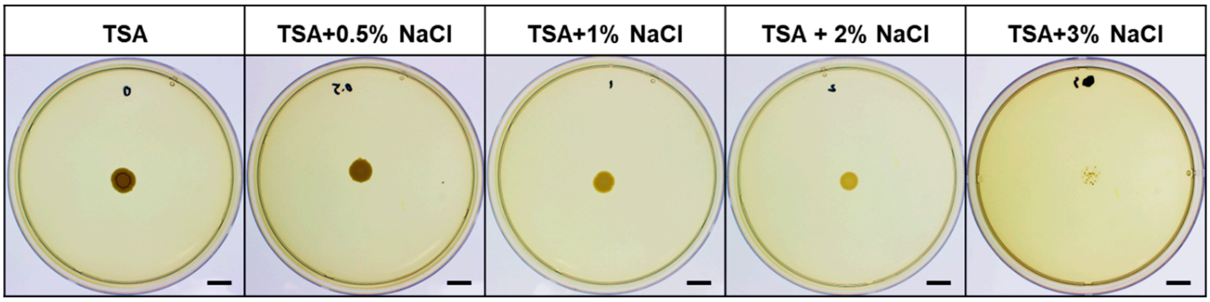

Figure S1. Comparison of *B. vietnamiensis* under different concentrations of NaCl.

B. vietnamiensis was incubated in different NaCl concentrations for 4 days. Representative images show colony size. Scale bars = 1 cm.
